# Regional tau pathology and loneliness in cognitively normal older adults

**DOI:** 10.1038/s41398-018-0345-x

**Published:** 2018-12-18

**Authors:** Federico d’Oleire Uquillas, Heidi I. L. Jacobs, Kelsey D. Biddle, Michael Properzi, Bernard Hanseeuw, Aaron P. Schultz, Dorene M. Rentz, Keith A. Johnson, Reisa A. Sperling, Nancy J. Donovan

**Affiliations:** 1 Department of Neurology, Massachusetts General Hospital, Harvard Medical School, Boston, MA 02114 USA; 2 Division of Nuclear Medicine, Department of Radiology, Massachusetts General Hospital, Harvard Medical School, Boston, MA 02114 USA; 3 School for Mental Health and Neuroscience, Alzheimer Centre, Limburg, Maastricht University, Maastricht, The Netherlands; 4 Department of Psychiatry, Division of Geriatric Psychiatry, Brigham and Women’s Hospital, Harvard Medical School, Boston, MA 02115 USA; 5 Department of Neurology, Saint-Luc University Hospital, Institute of Neuroscience, Université Catholique de Louvain, Brussels, Belgium; 6 Department of Neurology, Brigham and Women’s Hospital, Harvard Medical School, Boston, MA 02115 USA; 7 Department of Psychiatry, Massachusetts General Hospital, Harvard Medical School, Boston, MA 02114 USA

## Abstract

Loneliness is a perception of social and emotional isolation that increases in prevalence among older adults during the eighth decade of life. Loneliness has been associated with higher brain amyloid-β deposition, a biologic marker of Alzheimer’s disease, in cognitively normal older adults, suggesting a link with preclinical Alzheimer’s disease pathophysiology. This study examined whether greater loneliness was associated with tau pathology, the other defining feature of Alzheimer’s disease, in 117 cognitively normal older adults. Using flortaucipir positron emission tomography, we measured tau pathology in the entorhinal cortex, a region of initial accumulation in aging adults with or without elevated amyloid-β, and in the inferior temporal cortex, a region of early accumulation typically associated with elevated amyloid-β and memory impairment. Loneliness was measured by self-report using the 3-item UCLA-loneliness scale. We found that higher tau pathology in the right entorhinal cortex was associated with greater loneliness, controlling for age, sex, and apolipoprotein E ε4, the Alzheimer’s disease genetic risk marker. This association remained significant after further adjustment for socioeconomic status, social network, depression and anxiety scores, and memory performance. There was no association of inferior temporal cortical or left entorhinal tau pathology with loneliness. Exploratory whole-brain surface maps supported these findings and identified additional clusters correlating loneliness and tau in the right fusiform gyrus. These results provide further support for loneliness as a socioemotional symptom in preclinical Alzheimer’s disease.

## Introduction

The National Institute on Aging—Alzheimer’s Association Research Framework now defines Alzheimer’s disease (AD) as a pathologic continuum, a process of cerebral amyloid-β (Aβ) and tau accumulation that begins when individuals are cognitively unimpaired^1^. Increasing evidence suggests that transitional cognitive changes or neurobehavioral symptoms may be initial manifestations of this pathophysiologic process prior to the onset of overt cognitive impairment, a period of time also referred to as the “preclinical stage” of AD, according to earlier nomenclature^1–4^. Recognition of these initial neurobehavioral changes may provide unique insights into early AD pathogenesis and a greater opportunity for intervention prior to the development of more advanced neuropsychiatric morbidity and syndromal stages of cognitive and functional impairment.

Loneliness is a perception of social and emotional isolation that increases in prevalence among older adults during the eighth decade of life^5^. Loneliness is a subjective, but measurable construct that is distinct from objective social isolation^6^. In numerous studies, loneliness has been independently associated with subsequent declines in cognition and an increased risk of incident AD dementia in community-dwelling older adults^7–9^. These associations suggest that loneliness may be a specific risk factor for cognitive decline or an early neurobehavioral marker of AD pathological changes. Focusing on loneliness in a well-defined cohort of cognitively normal (CN) older adults, we considered that this subjective sense of social disconnection may be a very early symptom of Aβ deposition and tau pathology in regions involved in cognitive, emotional or perceptual processes important for social interactions.

We previously evaluated a sample of CN older adults and found that higher Pittsburgh Compound B (PiB)-positron emission tomography (PET) measures of neocortical Aβ deposition were associated with greater self-reported loneliness, assessed by the UCLA-loneliness scale, independent of sociodemographic factors, objective measures of social network, depressive, and anxiety symptoms^10^. This association was also stronger in carriers of the apolipoprotein E ε4 allele (APOEε4), the major genetic risk factor for sporadic, late-onset AD, compared to non-carriers^10^. Building on these findings we sought to further validate loneliness as a neurobehavioral symptom in preclinical AD in an enlarged sample. We hypothesized that symptoms of loneliness may also be associated with tau pathology in sites of initial tau accumulation during preclinical AD.

To test this, we examined whether higher levels of tau pathology in the entorhinal cortex (EC) and/or inferior temporal cortex (IT), determined by Flortaucipir (FTP)-PET, were associated with greater loneliness after adjusting for age, sex, APOEε4 status, socioeconomic status, social network, depression, anxiety, and memory function within this CN sample. We focused on two regions: the EC, a region of initial tau accumulation in aging adults without and with elevated Aβ, and the IT, a region of early accumulation typically associated with elevated Aβ and memory impairment^11,12^. We hypothesized that higher measures of tau pathology would be associated with greater loneliness in the EC, and possibly in the IT, consistent with loneliness as an early manifestation of AD pathophysiology.

## Methods

### Participants

One hundred and seventeen, community-dwelling, older-adult participants from the Harvard Aging Brain Study (HABS) were included. Participants were English-speaking men and women who were all cognitively normal based on Clinical Dementia Rating^13^ global score 0 and education-adjusted performance on the Wechsler Logical Memory subtest^14^ and the Mini-Mental State Examination (MMSE)^15^. Participants were free from active, major psychiatric disorders according to study exclusion criteria, as previously described^10^. All participants scored below cutoff for mild depression (less than or equal to 10) based on the 30-item Geriatric Depression Scale (GDS)^16^. Participants completed specialized instruments to assess loneliness, anxiety, and social network characteristics, and underwent both PiB and FTP-PET procedures. PiB-PET data from 62 of the 117 participants in this study were previously evaluated and reported^10^. The Partners Human Research Committee approved this study, and all participants provided written informed consent.

### Clinical measures

Loneliness was measured using the 3-item version of the UCLA-loneliness scale (UCLA-loneliness), a validated, self-rated instrument that has been implemented in numerous epidemiologic studies of aging^5,7,17,18^. The instrument asks, “How often do you feel you lack companionship? How often do you feel left out? How often do you feel isolated from others?” Each question was scored on a 4-point scale (item range: 1–4) corresponding to “never”, “rarely”, “sometimes”, or “often”, and the total loneliness score was calculated as the sum of these scores (range: 3–12, higher score indicating greater loneliness).

Seven items corresponding to anxiety symptoms from the 14-item hospital anxiety depression scale (HADS-anxiety)^19^ were used to calculate an anxiety score; each statement was rated for frequency (range: 0–3), with higher scores indicating greater anxiety (total score possible range: 0–21). Depression was calculated as the total score for the GDS (item score: 0–1; total score: 0–30; higher score indicating greater severity).

A social network score^20–23^ was calculated as the sum of 4 binary domain scores based on whether or not the study participant was (1) married, (2) had, in total, three or more friends, children, or relatives who visited monthly, (3) was a member of a community group, and (4) was a participant in religious services or activities (possible range: 0–4; higher score indicating greater social network).

Socioeconomic status was assessed using the Two-Factor Hollingshead score, pertaining to occupation and educational attainment (range: 11–65 in this sample, with higher score indicating lower socioeconomic status)^24^.

For these analyses, memory performance was defined by the logical memory-delayed recall score^14^ as assessed during the neuropsychiatric visit.

Specialized instruments for loneliness and all other clinical variables were administered to participants in a blinded fashion with regard to other assessments and procedures.

Participants were classified as either APOEε4 carriers or non-carriers based on genotype analyses.

### Flortaucipir (FTP) – positron emission tomography (PET)

FTP-PET imaging data were acquired on average 124.10 days [median = 110.0, interquartile range (IQR) = 69.0, 154.0, minimum = 2.0, maximum = 356.0, days] from the baseline clinical visit. Tau pathology was measured using Fluorine 18-FTP according to previously described methods^25^. FTP was acquired from 80 to 100 min after a 9.0 to 11.0 mCi bolus injection in 4 × 5-min frames. PET data were reconstructed and attenuation-corrected, and each frame was evaluated to verify adequate count statistics and absence of head motion. To evaluate the anatomy of cortical FTP binding, each individual PET dataset was rigidly coregistered to the subject’s MPRAGE data using SPM12 (Wellcome Department of Cognitive Neurology, Function Imaging Laboratory, London). The FreeSurfer (FS, version 6.0) regions of interest (ROIs) defined by MR as described above were transformed into the PET native space. PET data were partial volume-corrected (PVC) using the Geometrical Transfer Matrix (GTM) method as implemented in FreeSurfer or the extended Müller-Gartner for surface analyses^26^. FTP-specific binding was calculated from the right and left EC, and the right and left IT, as the standardized uptake value ratio (SUVr) using FS’s cerebellar gray ROI as reference. Right and left EC and IT regions were evaluated separately, as previous work has demonstrated a potential asymmetric vulnerability to tau pathology in the right hemisphere^25,27,28^.

### Pittsburgh compound-B (PiB)—positron emission tomography (PET)

PiB-PET imaging data were acquired on average 106.80 days (median = 113.0, IQR = 56.0, 140.0, minimum = 0, maximum = 428.0, days) from the neuropsychiatric visit, and on average 102.20 days (median = 56.0, IQR = 15.0, 139.0, minimum = 0, maximum = 484.0, days) from the acquisition of FTP-PET data. Aβ deposition was measured using PiB according to previously described methods^29^. Using Logan’s graphical analysis method^30^, we calculated PiB retention expressed as a distribution volume ratio (DVR) using a gray matter cerebellum reference region^31^ and PV-corrected using the GTM assuming a uniform 6-mm point spread function. The regions of interest for our meta-ROI, including the frontal, lateral parietal, lateral temporal, and retrosplenial cortices, were generated through FS and transformed to native-PET space^32^.

### Statistical analyses

Unadjusted associations between demographic and clinical variables and UCLA-loneliness scores were evaluated using Pearson correlations and linear regression.

In separate linear regression models, we first examined cross-sectional associations of FTP binding in either right EC, left EC, right IT, or left IT, with UCLA-loneliness as the dependent variable. Each of these primary models adjusted for age, sex, and APOEε4 carrier status and was corrected for multiple comparisons using the Bonferroni method.

For all models, residual-vs-fits plots, normality plots, and variance inflation factors were evaluated for each variable to ensure that distributions reasonably satisfied model assumptions.

To further assess the robustness of our findings, non-parametric equi-tailed bootstrapped confidence intervals were attained for all models using the adjusted bootstrap percentile interval with bias and acceleration correction found at the 0.025 and 0.975 quantiles of the corrected distribution, with a total of 5000 bootstrap replicates generated. Use of the bootstrap procedure provided a method to overcome potential limitations posed by right-tailed skews in the UCLA-loneliness or PET ligand binding measures that were significantly different from a normal distribution. This procedure also provided a means to construct a sampling distribution for our estimates, ensuring an accurate inference and calculation of 95% confidence intervals. Statistics for these models are reported in tables and include Cohen’s *f*^2^ effect sizes for the predictor of interest.

In a series of secondary models, we tested the specificity of the FTP binding and UCLA-loneliness association by adjusting for several factors that have been correlated with loneliness in prior studies of older adults, including socioeconomic status (Hollingshead), social network, depression (GDS), and anxiety (HADS-anxiety)^10,33^. We also adjusted for memory (Logical Memory-delayed recall) performance and time between neuropsychiatric and FTP-PET assessments. Each covariate was added separately to the main model to assess its individual influence and then all covariates were added to a comprehensive model.

Extending previously published findings, the cross-sectional association of PiB binding with UCLA-loneliness was also evaluated, adjusting for age, sex, and APOEε4 as in the primary models. This replication in an enlarged sample also tested whether APOEε4 carrier status modified the association of PiB binding and UCLA-loneliness, as previously shown^10^, by including an additional term for the multiplicative interaction of APOEε4 and PiB binding.

To investigate whether Aβ deposition and regional tau pathology contributed independently and/or synergistically to loneliness, we carried out secondary, exploratory models, including both PiB and FTP binding, without and with their multiplicative interaction, as predictors of UCLA-loneliness score.

In a post hoc model we examined whether APOEε4 carrier status modified the association of regional FTP binding with UCLA-loneliness identified in the main analysis.

### Exploratory whole-brain surface maps correlating loneliness and FTP binding

To visualize regional associations of tau pathology and loneliness more broadly, we calculated surface maps of correlations between FTP SUVr on every vertex of the surface with UCLA-loneliness score, adjusting for age, sex, and APOEε4 carrier status. These maps were generated by normalizing each participant’s native PET image to FS’s fsaverage surface and smoothing these images with an 8 mm Gaussian kernel. For comparison, a surface map adjusting for all additional covariates (age, sex, APOEε4 status, Hollingshead index, social network, GDS, and HADS-anxiety measures and Logical Memory-delayed recall z-scores) was performed (Figure S1). These correlation analyses were performed with in-house MATLAB scripts (http://mrtools.mgh.harvard.edu/).

Maps were performed as visualizations and as a tool to assess whether other regions not included in our a priori analyses should be assessed in future studies. We applied a liberal threshold of *p* < 0.01 (one-sided) consistent with this objective.

All statistical analyses employed R software (R, version 3.5; R Foundation for Statistical Computing, Vienna, Austria).

## Results

Demographic, clinical, and imaging data for these participants are reported in Table 1. The mean UCLA-loneliness score for the sample was 5.2, with scores comprising the full range of possible values (3–12). Overall, 21% of the sample (*n* = 24) endorsed feeling a lack of companionship “sometimes” or “often”, 17% (*n* = 20) endorsed feeling left out “sometimes” or “often,” and 13% (*n* = 15) endorsed feeling isolated “sometimes" or “often”. Among these participants, a small number endorsed “often”, the highest rating, for any of the three items [feeling a lack of companionship, (*n* = 2); feeling left out, (*n* = 2); feeling isolated, (*n* = 3)]. Most participants, 79% (*n* = 93) endorsed either “rarely” or “never” for all items. UCLA-loneliness scores in this sample were, therefore, mostly in the low to moderate range. UCLA-loneliness scores were positively correlated with GDS (*r* = 0.2, *p* = 0.04) and HADS-anxiety (*r* = 0.3, *p* = 0.003) scores, noting that depression scores for all participants were also in a subthreshold range. There was a negative, trend-level correlation of UCLA-loneliness scores with age (*r* = −0.2, *p* = 0.06), and no significant correlation of UCLA-loneliness with social network (*r* = −0.08, *p* = 0.4) or Hollingshead scores (*r* = 0.04, *p* = 0.7). Mean UCLA-loneliness scores did not differ across groups by sex (β = 0.03, *p* = 0.9), married/unmarried status (β = −0.61*, p* = 0.18) or APOEε4 carrier status (β = −0.13, *p* = 0.76).

**Table 1:** Demographic, clinical, and imaging characteristics of study participants

Characteristic	Value^a^	Range
*N* (total sample)	117	
Age (years)	76.01 (6.22)	64.75–92.25
Female, No. (%)	69 (59)	NA
Hollingshead score	25.61 (14.89)	11–65
Mini-mental state examination	29.25 (1.08)	25–30
UCLA-loneliness scale [range 3–12]	5.19 (1.95)	3–12
HADS anxiety-subscale [range 0–21]	10.84 (3.01)	7–19
Geriatric depression scale [range 0–30]	2.91 (2.43)	0–10
Social network score [range 0–4]	2.62 (0.88)	0–4
APOEε4 carrier status, positive, No. (%) (*n* = 112)	31 (28)	NA
Right entorhinal cortical FTP binding	1.28 (0.37)	0.36–2.99
Left entorhinal cortical FTP binding	1.38 (0.35)	0.39–2.56
Right inferior temporal cortical FTP binding	1.42 (0.20)	1.08–2.39
Left inferior temporal cortical FTP binding	1.50 (0.20)	1.12–2.65
Neocortical PiB binding	1.42 (0.44)	1.07–2.76
FTP to NP absolute time (days)	124.05 (80.93)	2.0–356.0
PiB to NP absolute time (days)	106.82 (69.64)	0.0–428.0

*NA* not applicable, *HADS* hospital anxiety and depression scale, *APOEε4* apolipoprotein E ε4, *FTP* flortaucipir, *PiB* pittsburgh compound B, *NP* neuropsychiatric visit

^a^Unless otherwise indicated, data for 117 participants are shown and are reported as mean values with standard deviation

### Entorhinal and inferior temporal tau and loneliness

**Fig. 1:**
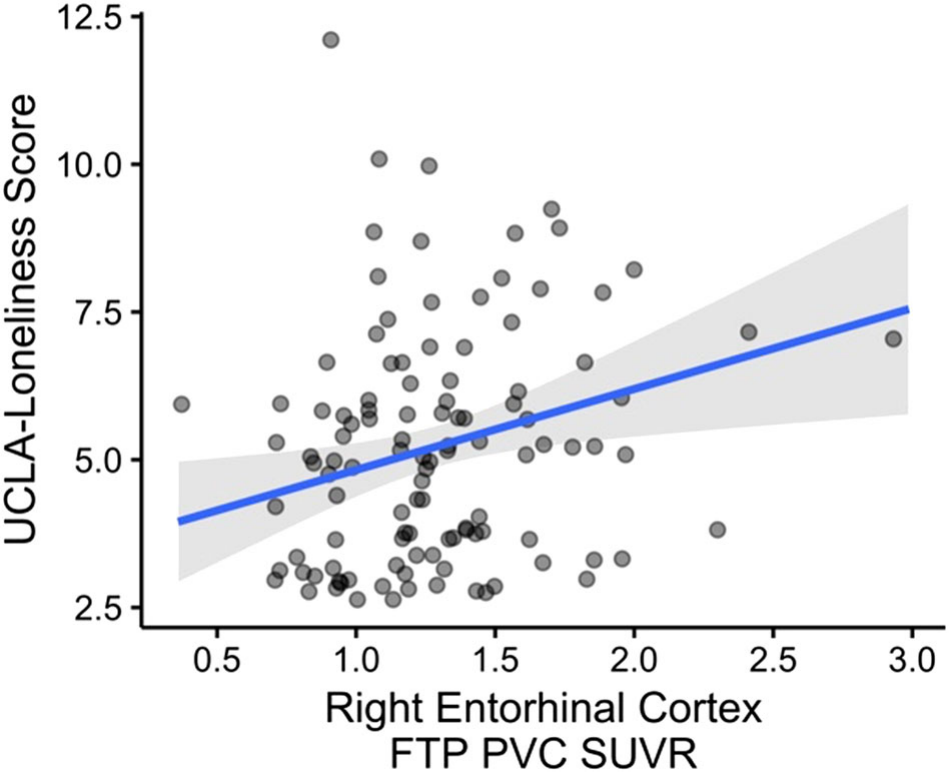
Multivariable regression was performed for UCLA-loneliness scores, the dependent variable (higher score indicates greater loneliness). Plotted are the estimated marginal means for Flortaucipir (FTP) partial volume-controlled (PVC) signal uptake ratio (SUVr) binding for the right entorhinal cortex. Covariates included age, sex, and apolipoprotein E e4 carrier status. For right entorhinal FTP binding: β = 1.37, 95% CI [0.28, 2.15], Cohen’s *f*^2^ = 0.254

In the first of four primary models, greater FTP binding in the right EC was associated with higher UCLA-loneliness scores, adjusting for age, sex, and APOEε4 carrier status, an association that remained significant after Bonferroni correction (Table 2, Fig. 1). There was no association of FTP binding with UCLA-loneliness scores in other regions (left EC, right IT and left IT) (Table 2).

**Table 2:** Primary analyses for the association of UCLA-loneliness with entorhinal cortical FTP binding, and inferior temporal cortical FTP binding

		Dependent variable: UCLA-loneliness scale			
		Left entorhinal cortex model	Right entorhinal cortex model	Left inferior temporal cortex model	Right inferior temporal cortex model
Age	β	−0.40	−0.46	−0.36	−0.45
	SE	(0.20)	(0.19)	(0.20)	(0.20)
	*p*-value	0.047*	0.018*	0.074	0.029*
Sex	β	0.04	0.15	0.02	0.07
	SE	(0.38)	(0.37)	(0.39)	(0.38)
	*p*-value	0.913	0.682	0.954	0.851
APOEε4 carrier status	β	−0.29	−0.53	−0.22	−0.38
	SE	(0.43)	(0.43)	(0.43)	(0.43)
	*p*-value	0.500	0.215	0.605	0.378
FTP binding	β	0.56	1.37	0.33	1.56
	SE	(0.58)	(0.52)	(1.01)	(1.03)
	*p*-value	0.337	0.010*	0.743	0.135
	95% BCa CI	[−0.61, 1.51]	[0.28, 2.15]	[−1.78, 1.89]	[−0.62, 3.59]
	Cohen’s *f*^2^	0.093	0.254	0.032	0.146
	Model adjusted R^2^	0.003	0.055	−0.005	0.015

Four models show the association of FTP binding in four a priori regions of interest with scores for the UCLA-loneliness scale, controlling for age, sex, and APOEε4 carrier statu. Statistics are presented as unstandardized estimate coefficient (β), standard error of the mean (SE), and *p*-value. Covariates were centered. p* < 0.05

*APOEε4* apolipoprotein E e4, *FTP* flortaucipir, *BCa CI* bias-corrected and accelerated bootstrapped confidence interval

The association of greater FTP binding in the right EC with higher UCLA-loneliness scores remained significant after adjustment for each of the psychosocial, neuropsychiatric, and cognitive covariates (Table 3). In addition to right EC FTP, younger age, higher GDS, and higher HADS-anxiety scores were other significant predictors of UCLA-loneliness scores in these models (Table 3). The association of right EC FTP binding and UCLA-loneliness remained significant in a secondary model that included all covariates: age, sex, APOEε4, Hollingshead, social network, GDS, HADS-anxiety, Logical Memory-delayed recall scores and time between visits (for right EC FTP: β = 1.22, *p* = 0.021, [0.27, 2.09], Cohens *f*^2^ *=* 0.200).

**Table 3:** Secondary analyses for the association of UCLA-loneliness scale with entorhinal cortical FTP binding after adjustment for additional covariates

		Dependent variable: UCLA-loneliness scale					
		Adjusting for hollingshead index	Adjusting for social network	Adjusting for geriatric depression scale	Adjusting for HADS-anxiety scale	Adjusting for time difference between visits	Adjusting for logical memory-delayed recall
Age	β	−0.46	−0.47	−0.60	−0.42	−0.45	−0.46
	SE	(0.19)	(0.19)	(0.19)	(0.19)	(0.19)	(0.19)
	*p*-value	0.020*	0.017*	0.003**	0.025*	0.022*	0.020*
Sex	β	0.15	0.15	0.26	0.38	0.14	0.21
	SE	(0.38)	(0.37)	(0.37)	(0.37)	(0.38)	(0.39)
	*p*-value	0.702	0.695	0.473	0.308	0.718	0.584
APOEε4 carrier status	β	−0.52	−0.48	−0.46	−0.43	−0.53	−0.55
	SE	(0.43)	(0.43)	(0.42)	(0.41)	(0.43)	(0.43)
	*p*-value	0.227	0.267	0.273	0.304	0.215	0.201
Additional covariate	β	−0.03	−0.11	0.49	0.53	0.12	0.03
	SE	(0.19)	(0.19)	(0.19)	(0.18)	(0.18)	(0.05)
	*p*-value	0.858	0.541	0.012*	0.004**	0.504	0.550
Right EC FTP binding	β	1.36	1.33	1.26	1.29	1.38	1.40
	SE	(0.53)	(0.53)	(0.51)	(0.51)	(0.52)	(0.53)
	*p*-value	0.012*	0.014*	0.016*	0.012*	0.010*	0.009**
	95% BCa CI	[0.25, 2.14]	[0.27, 2.11]	[0.22, 2.05]	[0.20, 2.03]	[0.25, 2.21]	[0.30, 2.22]
	Cohen’s *f*^2^	0.251	0.245	0.240	0.249	0.257	0.259
	Model adjusted *R*^2^	0.046	0.049	0.102	0.118	0.050	0.049

Models show how the association of right entorhinal cortex flortaucipir binding and UCLA-loneliness scale scores is influenced, separately, by each additional covariate. Statistics are presented as the unstandardized estimate coefficient (β), standard error of the mean (SE), and *p*-value. Covariates were centered. p* < 0.05, p** < 0.01

*EC* entorhinal cortex, *APOEε4* apolipoprotein E e4, *FTP* flortaucipir, *BCa CI* bias-corrected and accelerated bootstrapped confidence interval

To address the possibility of an outlier effect, the main model was refit after removal of the highest value for right EC FTP (a statistical outlier per Grubb’s test), or the highest two values. In both cases, the association of right EC FTP binding with UCLA-loneliness scores remained significant (Supplement Table S1).

Similar results were obtained for the association of right EC FTP and UCLA-loneliness scores when restricting the sample to participants with maximum 6 or 9-month (rather than 12 months) time delays between FTP-PET and neuropsychiatric assessments (Supplement Table S2). Results were unchanged using pre- or post PVC data (not shown).

In a post hoc model, we found that APOEε4 carrier status did not modify the association of right entorhinal FTP and UCLA-loneliness scores (for the interaction of right EC FTP and APOEε4: β = 0.52, *p* = 0.60).

### Neocortical amyloid-β and loneliness

Greater PiB binding was associated with higher UCLA-loneliness scores, adjusting for age, sex and APOEε4, and the association was stronger in APOEε4 carriers compared to non-carriers (Supplement Table S3).

### Neocortical amyloid-β, tau, and loneliness

When right EC FTP and neocortical PiB binding were included together as main terms, right EC FTP binding was marginally associated with UCLA-loneliness scores and the association of PiB binding with UCLA-loneliness scores was non-significant (right EC FTP: β = 1.07, *p* = 0.071, Cohen’s *f*^2^ = 0.255; PiB: β = 0.61, *p* = 0.252, Cohen’s *f*^2 ^= 0.112). The multiplicative interaction of right EC FTP and PiB binding was not associated with UCLA-loneliness scores (for right EC FTP × PiB: β = −0.82, *p* = 0.439).

### Exploratory whole-brain surface maps correlating loneliness and FTP binding

**Fig. 2:**
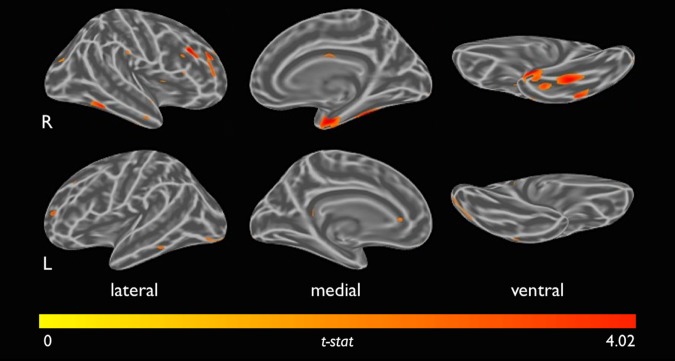
Shown above is the vertex-wise map correlating UCLA-loneliness scores with Flortaucipir signal uptake ratio, covaried for age, sex and apolipoprotein E e4 carrier status. PET data were partial volume-corrected using the extended Müller-Gartner correction for surface analysis. Red colors indicate positive associations and reflect the one-sided *t*-statistic with threshold *t* > 2.36 (*p* = 0.01)

Whole-brain, vertex-wise correlations were consistent with our region-of-interest results, showing correlations of UCLA-loneliness scores with FTP binding in the right EC (*p* < 0.01), co-varying for age, sex, and APOEε4 status (Fig. 2). Maps revealed additional clusters in the right inferior temporal cortex and right fusiform gyrus (Fig. 2). For comparison, a map with all additional covariates (age, sex, APOEε4 status, Hollingshead index, social network, GDS and HADS-anxiety measures and Logical Memory-delayed recall z-scores) showed a similar pattern with more prominent UCLA-loneliness scores and FTP binding in the same regions (Supplement Fig. 1).

## Discussion

The goal of this study was to examine loneliness as an initial and prototypical neurobehavioral symptom in early AD by assessing its association with brain tau pathology in cognitively normal older adults. We found that higher tau pathology in the right entorhinal cortex was associated with more frequent feelings of lacking companionship, feeling left out or feeling isolated. Consistent with earlier published findings from a smaller sample, higher Aβ burden was also associated with greater loneliness, particularly in carriers of the APOEε4 allele, the major genetic risk factor for late-onset AD^10^. Together, these findings support the hypothesis that loneliness may be a sensitive subjective marker of very early AD pathological changes.

Our use of the three-item version of the UCLA-loneliness scale harmonizes with a number of international cohort studies of aging^5,6,34,35^. Using this instrument, population-based, prospective studies have found that greater loneliness predicted cognitive and functional declines, independent of sociodemographic factors, depression, anxiety, and social network size^6,7,34^. Compared to most studies that included participants with heterogeneous cognitive function, levels of depressive symptoms and mental health histories, we focused on a more select sample of participants with strictly defined normal cognition and an absence of mild depression and other psychiatric conditions. Elevated loneliness ratings in this study were generally intermediate in severity and were endorsed by only 21% of the sample. By comparison, Perissinotto and colleagues reported that 30% of participants from the nationally representative, U.S. Health and Retirement Study, ages 60 and older, endorsed loneliness symptoms of equivalent (moderate) severity, and 13% endorsed more severe symptoms^34^. Importantly, they found that participants classified as both “moderately” and “severely” lonely, demonstrated similar adverse functional outcomes and higher risk of death compared to the non-lonely group^34^. This suggests that older adults with even moderate loneliness symptoms, as reported here, are a clinically meaningful group and may be on a continuum with those who are severely lonely. Our findings reveal that these moderate loneliness symptoms may be specifically associated with early Aβ and tau deposition in the earliest regions of pathological progression.

We chose the EC and IT as regions of interest for this study based on neuropathological staging and earlier in vivo PET studies that have described patterns of tau pathology in CN, mild cognitive impairment and AD dementia groups^11,12,36,37^. The EC is an initial site of tau pathology in typical aging as well as in early AD pathogenesis^38^. With widely distributed efferents to neighboring regions and to association and limbic cortices, the EC is a nexus between the hippocampus and neocortex that is critical for episodic and semantic memory processes^37,39^. This critical function includes information and memory processing with social and emotional content^40,41^. The EC is densely innervated by inhibitory serotonergic projections from the dorsal raphe nuclei^42^, and along with benzodiazepine, opiate, and neurotensin receptors, serotonin (5-HT2) receptors within the EC have been shown to be selectively depleted in AD dementia^43^. We reasoned that higher levels of tau-related neurodegeneration within the EC might disrupt functional connections with nearby limbic and neocortical regions and be associated with altered socioemotional processes corresponding to greater loneliness. The IT was chosen as a second, contrasting, region of interest, as it is a locus of early tau progression in AD beyond the EC. Higher IT tau pathology has been associated with elevated Aβ burden and lower performance on memory testing, and is more closely associated with syndromal stages of AD such as mild cognitive impairment^12^.

In the main analyses for the current study, we found that loneliness was associated with EC but not IT tau pathology, although small clusters of correlations of loneliness with IT tau were found in the whole-brain map. These differential effects are consistent with a regional-functional dissociation such that EC tau binding is more closely associated with loneliness symptoms, whereas IT tau binding is associated with declines in memory function^12^. This interpretation is further supported by the secondary model showing that the association of EC tau binding and loneliness was not altered by objective measures of memory. In prior work, Gatchel et al. evaluated a sample of CN older adults which included participants with mild and moderate levels of depression^44^. GDS score was weakly associated with bilateral IT tau and marginally with EC tau pathology, adjusting for sex^44^. This is in contrast to our non-depressed sample, in which we find a more specific association of loneliness with right EC tau, and possibly right fusiform tau, independent of age, sex, APOEε4, GDS and many other factors. These results reveal that tau pathology in the right EC was associated with a dimension of loneliness unrelated to known psychosocial and neuropsychiatric confounders, suggesting a distinct underlying neural mechanism.

Exploratory maps revealed an unexpected localization of greater loneliness and higher tau pathology in the right fusiform gyrus. In recent work, Jack and colleagues identified the fusiform gyrus and posterior cingulate cortex as regions of tau pathology that best distinguished cognitively normal participants with high levels of Aβ from those with low Aβ, and defined these combined regions as the early Alzheimer’s disease change meta-region of interest^45^. In this context, associations of loneliness with higher cortical Aβ and higher tau in the fusiform gyrus point to loneliness as a potential marker of more advanced, and possibly, more active AD pathophysiology in cognitively normal older adults.

Tau pathology and neurodegeneration in fusiform and entorhinal regions could be associated with altered face processing or deficiencies in the retrieval of semantic information from faces^46–48^. Previous studies have shown age-related differences in brain activation during emotional face processing and face recognition tasks using functional MRI^46,47^. Unlike younger adults, older adults failed to activate right temporal and limbic regions during emotional face processing, including the fusiform gyrus, but recruited other regions, suggesting compensatory network reorganization. Whereas the etiology of this functional reorganization in normal older adults is unknown, it is possible that AD pathological changes in the EC and functionally connected brain regions, particularly in the right hemisphere, may contribute to appreciable deficiencies in the processing of social interactions and reduce the rewarding qualities of social engagement.

A limitation of this study is that we measured loneliness at a single time point and did not assess the onset or chronicity of loneliness. Further, these cross-sectional analyses do not address the directionality of the associations of loneliness with AD biomarkers. It is possible that chronic loneliness in the context of socioemotional deficits or personality factors, such as neuroticism, may potentiate AD pathophysiology. While the selectivity of this sample allowed us to examine the loneliness construct from a new perspective, this also limits the generalizability of our findings. Few study participants endorsed high levels of loneliness, therefore, these analyses did not address whether severe loneliness and/or loneliness in the context of clinical depression is associated with similar patterns of regional tauopathy or higher Aβ deposition. It is also possible that the association of loneliness with tau pathology in the right, but not left hemisphere was influenced by selection bias due to screening procedures that excluded participants based on cognitive performance thresholds. Finally, as Aβ and tau were relatively restricted in range and were highly correlated, a larger sample with a wider range of values for Aβ and tau may be necessary to resolve whether Aβ and tau burden interactively relate to loneliness.

In conclusion, we found that higher tau pathology in the right entorhinal cortex was associated with more frequent feelings of loneliness in CN older adults without clinically significant depression. Extending prior work, we again showed an association of higher neocortical Aβ burden with greater loneliness. These findings point to associations of loneliness with right-sided tau pathology in the earliest sites of AD pathogenesis, the entorhinal cortex, and possibly the fusiform gyrus. Subjective feelings of social detachment may accompany very early AD pathological changes in brain regions relevant to social function in CN older adults.

## Supplementary information

Supplemental Material
