# Supplementary material for: Regional tau pathology and loneliness in cognitively normal older adults
Source: Transl Psychiatry. 2018 Dec 18;8:282. doi: 10.1038/s41398-018-0345-x (PMC6299114; doi:10.1038/s41398-018-0345-x)
Supplement: Supplementary file 1 — Supplemental Material [file 41398_2018_345_MOESM1_ESM.pdf]

Supplemental Figures and Tables

d'Oleire Uquillas F, Jacobs HIL, Biddle KD, Properzi MR, Hanseeuw BJ, Schultz AP, Rentz DM, Johnson KA, Sperling RA, Donovan NJ. Regional Tau Pathology and Loneliness in Cognitively Normal Older Individuals

Contents:

Supplemental Figure 1. Vertex-Wise Map Correlating UCLA-Loneliness Scale scores with FTP Binding, adjusting for age, sex, APOEε4 carrier status, psychosocial, neuropsychiatric and cognitive covariates and time between PET scan and neuropsychiatric visit

Supplemental Table S1. Secondary Models for the Association of UCLA-Loneliness Scale and Right Entorhinal FTP Binding without Extreme Values

Supplemental Table S2. Secondary Model for the Association of UCLA-Loneliness Scale and Right Entorhinal FTP Binding with Greater Restriction for Time Delay Between Visits

Supplemental Table S3. Models for the Association of UCLA-Loneliness with PiB Binding without and with its Multiplicative Interaction with APOE ε4 Carrier Status

Supplemental Table S4. Secondary Models for the Association of Cognition and Right and Left Entorhinal FTP Binding and Inferior Temporal Binding

Supplemental Figure 1: Vertex-Wise Map Correlating UCLA-Loneliness Scale scores with FTP Binding, adjusting for age, sex, APOEε4 carrier status, psychosocial, neuropsychiatric and cognitive covariates and time between PET scan and neuropsychiatric visit.

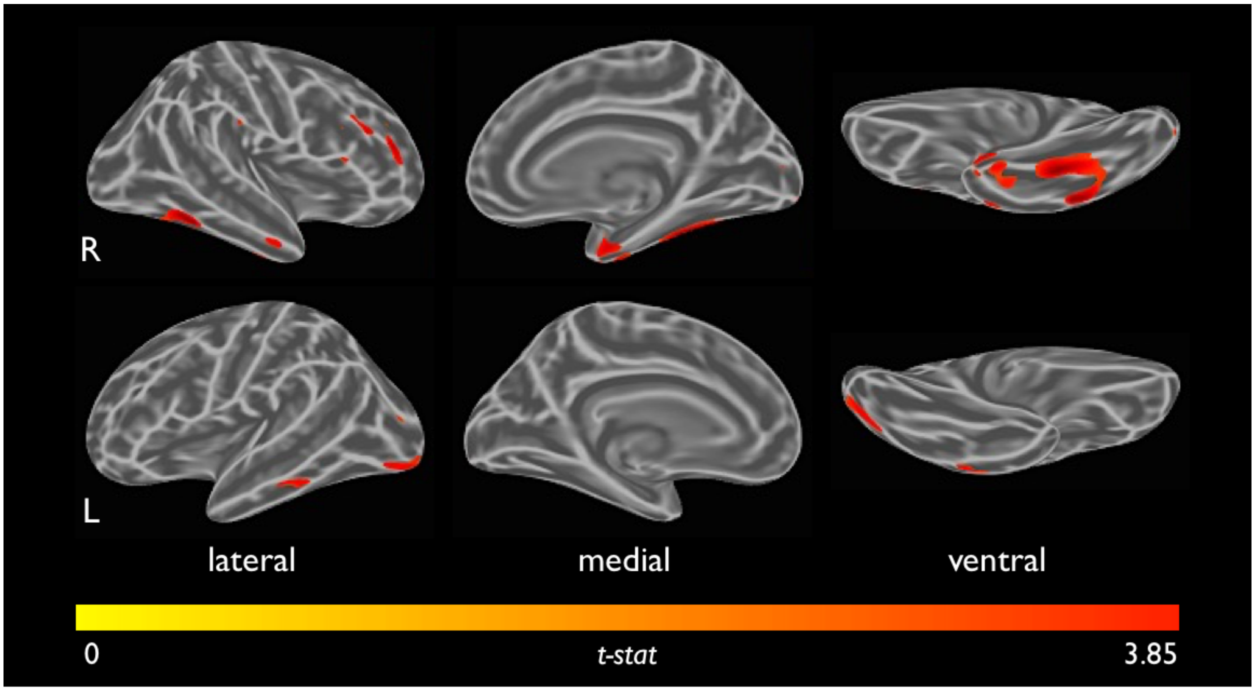

Supplemental Fig. S1. Flortaucipir signal uptake ratio (FTP SUVr) PET data were partial volume-corrected using the extended Müller-Gartner correction for surface analysis. Vertex-wise map shows the association of FTP SUVr with scores for the UCLA-loneliness Scale, covaried for age, sex, APOEε4 carrier status, socioeconomic status, social network score, depression, anxiety, logical memory delayed recall performance and time between PET scan and neuropsychiatric visit. Red colors indicate positive associations, and they reflect the one-sided t-statistic with threshold $t > 2.36$ ($p = 0.01$).

Supplemental Table S1: Secondary Models for the Association of UCLA-Loneliness Scale and Right Entorhinal FTP Binding without Extreme Values

		Dependent variable: **UCLA-Loneliness Scale**	
		Without Statistical Outlier	*Without 2 Most Extreme Values*
Age	β	-0.47	-0.47
	SE	(0.19)	(0.19)
	p-value	**0.016***	**0.017***
Sex	β	0.16	0.16
	SE	(0.37)	(0.38)
	p-value	0.673	0.667
APOEε4 Carrier Status	β	-0.54	-0.52
	SE	(0.43)	(0.43)
	p-value	0.209	0.230
Right EC FTP Binding	β	1.28	1.23
	SE	(0.56)	(0.61)
	p-value	**0.025***	**0.045***
	95% BCa C.I.	[0.07, 2.23]	[0.01, 2.35]
	Cohen's f^2	0.221	0.198
	Model Adjusted R^2	0.049	0.042

Models show the association of FTP binding in the right entorhinal cortex (EC) with scores for UCLA-loneliness Scale, controlling for age, sex, and APOEε4 carrier status, after removing a statistical outlier value for right EC FTP (left), and without the 2 most extreme values for right EC FTP binding, respectively (right).
Abbreviations: APOEε4, apolipoprotein E e4; FTP, flortaucipir; BCa C.I., bias-corrected and accelerated bootstrapped confidence interval.
Covariates were centered. Statistics for each predictor are presented as unstandardized estimate coefficient (β), standard error of the mean (SE), and *p*-value. $p^* < 0.05$.

Supplemental Table S2: Secondary Model for the Association of UCLA-Loneliness Scale and Right Entorhinal FTP Binding with Greater Restriction for Time Delay Between Visits

		Dependent variable: **UCLA-Loneliness Scale**	
		6 Month Maximum Time Delay Restriction	*9 Month Maximum Time Delay Restriction*
Age	β	-0.32	-0.32
	SE	(0.22)	(0.20)
	p-value	0.140	0.113
Sex	β	-0.11	-0.06
	SE	(0.44)	(0.40)
	p-value	0.798	0.876
APOEε4 Carrier Status	β	-0.41	-0.38
	SE	(0.50)	(0.45)
	p-value	0.416	0.396
Right EC FTP Binding	β	1.15	1.08
	SE	(0.59)	(0.54)
	p-value	0.052	**0.048***
	95% BCa C.I.	[0.10, 2.12]	[0.14, 2.06]
	Cohen's f^2	0.212	0.203
	Model Adjusted R^2	0.016	0.016

Models show the association of FTP binding in the right entorhinal cortex (EC) with scores for the UCLA-loneliness Scale, controlling for age, sex, and APOEε4 carrier status, after restricting data to conform to a 6-month maximum time delay between clinical and imaging visit, and after restricting data to a 9-month maximum time in-between delay, respectively.
Abbreviations: APOEε4, apolipoprotein E e4; FTP, flortaucipir; BCa C.I., bias-corrected and accelerated bootstrapped confidence interval.
Covariates were centered. Statistics for each predictor are presented as unstandardized estimate coefficient (β), standard error of the mean (SE), and *p*-value. $p^{*}<0.05$.

Supplemental Table S3: Models for the Association of UCLA-Loneliness with PiB Binding without and with its Multiplicative Interaction with APOE ε4 Carrier Status

		Dependent variable: UCLA-Loneliness Scale	
Age	β	-0.43	-0.39
	SE	(0.19)	0.19
	p-value	**0.028***	**0.041***
Sex	β	0.06	0.15
	SE	(0.37)	0.37
	p-value	0.868	0.691
APOEε4 Carrier Status	β	-0.68	-3.94
	SE	(0.47)	1.46
	p-value	0.145	**0.008****
PiB Binding	β	1.04	-0.09
	SE	(0.47)	0.67
	p-value	**0.030***	0.895
	95% BCa C.I.	[0.18, 1.76]	[-1.39, 1.14]
	Cohen's f^2	0.212	0.217
PiB Binding x APOEε4 Status	β		2.15
	SE		0.92
	p-value		**0.021***
	95% BCa C.I.		[0.59, 3.61]
	Cohen's f^2		0.228
	Model Adjusted R^2	0.037	0.019

Models show associations of Pittsburgh Compound-B (PiB) binding, and the interaction of PiB binding by APOEε4 carrier status with scores for the UCLA-loneliness Scale, controlling for age, sex, and APOEε4 carrier status.
Abbreviations: APOEε4, apolipoprotein E e4; BCa C.I., bias-corrected and accelerated bootstrapped confidence interval.
Covariates were centered. Statistics for each predictor are presented as unstandardized estimate coefficient (β), standard error of the mean (SE), and *p*-value. $p^{*}<0.05$, $p^{**}<0.01$.

Supplemental Table S4: Secondary Models for the Association of Cognition and Right and Left Entorhinal FTP Binding and Inferior Temporal Binding

		Dependent variable: **Preclinical Alzheimer Cognitive Composite**			
		Left EC FTP Binding Model	*Right EC FTP Binding Model*	*Left IT FTP Binding Model*	*Right IT FTP Binding Model*
Age	β	-0.24	-0.23	-0.24	-0.21
	SE	(0.07)	(0.07)	(0.07)	(0.07)
	p-value	**0.001****	**0.001****	**0.001****	**0.003****
Sex	β	-0.07	-0.08	-0.07	-0.09
	SE	(0.13)	(0.13)	(0.13)	(0.13)
	p-value	0.590	0.529	0.582	0.498
APOEε4 Carrier Status	β	-0.001	0.03	-0.001	0.05
	SE	(0.15)	(0.15)	(0.15)	(0.15)
	p-value	0.997	0.860	0.995	0.744
Pathology Marker	β	-0.04	-0.14	-0.09	-0.48
	SE	(0.20)	(0.19)	(0.35)	(0.36)
	p-value	0.831	0.451	0.808	0.191
	95% BCa C.I.	[-0.41, 0.30]	[-0.60, 0.23]	[-0.82, 0.69]	[-1.35, 0.31]
	Cohen's f^2	0.021	0.073	0.024	0.127
	Model Adjusted R^2	0.083	0.088	0.083	0.098

Models show associations of left or right entorhinal cortex (EC) flortaucipir (FTP) binding, or left or right inferior temporal (IT) FTP binding with Preclinical Alzheimer Cognitive Composite *z*-scores, controlling for age, sex, and APOEε4 carrier status.
Abbreviations: APOEe4, apolipoprotein E e4; FTP, flortaucipir; BCa C.I., bias-corrected and accelerated bootstrapped confidence interval.
Covariates were centered. Statistics for each predictor are presented as unstandardized estimate coefficient (β), standard error of the mean (SE), and *p*-value. $p^{*}<0.05$, $p^{**}<0.01$.
